# Overall Water Splitting by a SrTaO_2_N-Based Photocatalyst Decorated with an Ir-Promoted Ru-Based Cocatalyst

**DOI:** 10.1021/jacs.2c11025

**Published:** 2023-01-20

**Authors:** Kaihong Chen, Jiadong Xiao, Junie Jhon M. Vequizo, Takashi Hisatomi, Yiwen Ma, Mamiko Nakabayashi, Tsuyoshi Takata, Akira Yamakata, Naoya Shibata, Kazunari Domen

**Affiliations:** † Research Initiative for Supra-Materials, Interdisciplinary Cluster for Cutting Edge Research, Shinshu University, Nagano-shi, Nagano 380-8553, Japan; ‡ PRESTO, JST, 4-17-1 Wakasato, Nagano-shi, Nagano 380-8553, Japan; § Institute of Engineering Innovation, The University of Tokyo, 2-11-16 Yayoi, Bunkyo-ku, Tokyo 113-8656, Japan; ∥ Graduate School of Natural Science & Technology, Okayama University, 3-1-1 Tsushima-naka, Okayama 700-8530, Japan; ⊥ Office of University Professors, The University of Tokyo, 2-11-16 Yayoi, Bunkyo-ku, Tokyo 113-8656, Japan

## Abstract

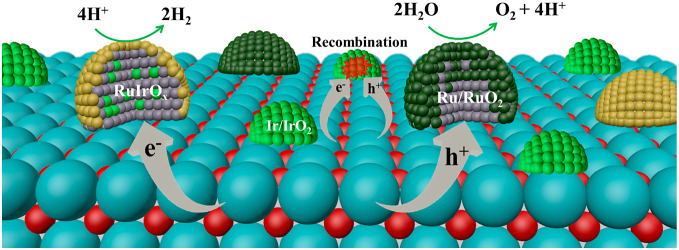

The development of narrow-bandgap photocatalysts for one-step-excitation overall water splitting (OWS) remains a critical challenge in the field of solar hydrogen production. SrTaO_2_N is a photocatalytic material having a band structure suitable for OWS under visible light (λ ≤ 600 nm). However, the presence of defects in the oxynitride and the lack of cocatalysts to promote simultaneous hydrogen and oxygen evolution make it challenging to realize OWS using this material. The present work demonstrates a SrTaO_2_N-based particulate photocatalyst for OWS. This photocatalyst, which was composed of single crystals, was obtained by nitriding SrCl_2_ and Ta_2_O_5_ together with NaOH, with the latter added to control the formation of defects. The subsequent loading of bimetallic RuIrO_*x*_ nanoparticles accelerated charge separation and allowed the SrTaO_2_N photocatalyst to exhibit superior OWS activity. This research presenting the strategies of controlling the oxygen sources and promoting the cocatalyst function is expected to expand the range of potential OWS-active oxynitride photocatalysts and permit the design of efficient cocatalysts for photocatalytic OWS.

One-step-excitation overall water splitting (OWS) using sunlight and a particulate photocatalyst offers a simple route to the realization of sustainable hydrogen production.^1,2^ Recently, solar hydrogen production on the 100 m^2^ scale has been achieved based on OWS with an Al-doped SrTiO_3_ particulate photocatalyst, thus demonstrating the feasibility of this process on a large scale.^3^ Nevertheless, the solar-to-hydrogen (STH) energy conversion efficiency of this 100 m^2^ panel system was only approximately 0.76%.^3^ In fact, increasing the STH efficiency of such systems beyond 1.7% is considered impossible because the photocatalyst responds solely to ultraviolet light.^1^ Obtaining an STH efficiency of 5%, which is the lowest value at which this process becomes practical, will require the development of the OWS photocatalysts having band gap energies, *E*_g_, below 2.1 eV.^1^ To date, InGaN-based nanorod arrays directly grown on a substrate have demonstrated STH of 3–5%.^4,5^ However, only a few particulate photocatalysts, such as LaMg_1/3_Ta_2/3_O_2_N,^6,7^ Ta_3_N_5_,^8^ Y_2_Ti_2_O_5_S_2_,^9^ and BaTaO_2_N,^10^ have been reported to have *E*_g_ ≤ 2.1 eV and to drive OWS, and the apparent quantum yield (AQY) under visible light was at most 0.36%.^9^

SrTaO_2_N (*E*_g_ = 2.1 eV) is a perovskite-type oxynitride photocatalyst with a band structure suitable for promoting both the hydrogen evolution reaction (HER) and oxygen evolution reaction (OER) from aqueous solutions under visible light. To date, SrTaO_2_N has been applied to drive either the HER or OER individually in the presence of sacrificial reagents^11,12^ but has not been successfully applied to OWS. This is partly due to the inevitable presence of defects in SrTaO_2_N that enhance charge recombination. Another crucial factor is the lack of cocatalysts to promote charge separation and simultaneous HER and OER. The dual cocatalyst strategy in which both a hydrogen evolution cocatalyst (HEC) and oxygen evolution cocatalyst (OEC) are incorporated into the OWS system has become popularly investigated to promote OWS effectively.^10,13,14^ However, the possible HEC candidates are presently limited to certain noble metals.^2^ Bimetals and bimetallic oxides may provide enhanced activities compared with monometallic cocatalysts owing to the tunability of physicochemical properties.^15,16^ Therefore, it would be desirable to explore the design of simple yet effective methods for synthesizing SrTaO_2_N with few defects and loading bimetallic metal or metal-oxide nanoparticles on photocatalyst surfaces jointly.

The present work demonstrates the application of a SrTaO_2_N-based particulate photocatalyst to promote OWS. The direct nitridation of SrCl_2_ and Ta_2_O_5_ in the presence of NaOH produced single-crystal SrTaO_2_N particles with few defects and byproducts. Loading nanoparticles of the bimetallic RuIrO_*x*_ with the CrO_*y*_ shell on this material allowed the SrTaO_2_N photocatalyst to exhibit superior OWS activity compared with other perovskite oxynitride photocatalysts.

SrTaO_2_N was synthesized by the nitridation of SrCl_2_, Ta_2_O_5_, and NaOH combined in a 4:1:*n* molar ratio. Here, the SrCl_2_ served as the Sr source as well as the flux, while the NaOH served as the O source and the flux. This material is referred to herein as SrTaO_2_N(*n*), where *n* is the NaOH/Ta_2_O_5_ molar ratio.

The X-ray diffraction (XRD) patterns in Figure 1A indicate that a combination of SrTaO_2_N and Ta_3_N_5_ phases was formed in the case of SrTaO_2_N(0). The amount of Ta_3_N_5_ in the product was decreased when adding NaOH during the synthesis of the SrTaO_2_N(1) specimen. Additional characterization by inductively coupled plasma-atomic emission spectroscopy (ICP-AES) and X-ray photoelectron spectroscopy (XPS) indicated that SrTaO_2_N(1) was not doped with Na (Figure S1a). The weight fractions of Sr, Ta, O, and N were measured to be 23.0, 61.1, 5.3, and 8.5%, respectively, indicating that the sample consisted of 0.78(SrTaO_2_N) + 0.07(Ta_3_N_5_). The data acquired using UV–visible diffuse-reflectance spectroscopy (DRS) as presented in Figure 1B and Figure S2B demonstrate that the optical absorption edge for each SrTaO_2_N(*n*) sample was at approximately 600 nm regardless of the value of *n*. An absorption background associated with defects in the photocatalyst is also observed beyond the absorption edge. Notably, these features related to defects and byproducts became weaker with an increasing amount of NaOH, and pure SrTaO_2_N was obtained at *n* = 2 (Figure S2). These results suggest that adding appropriate amounts of NaOH can reduce the defect density of SrTaO_2_N and the amount of the Ta_3_N_5_ byproduct.

**Figure 1:**
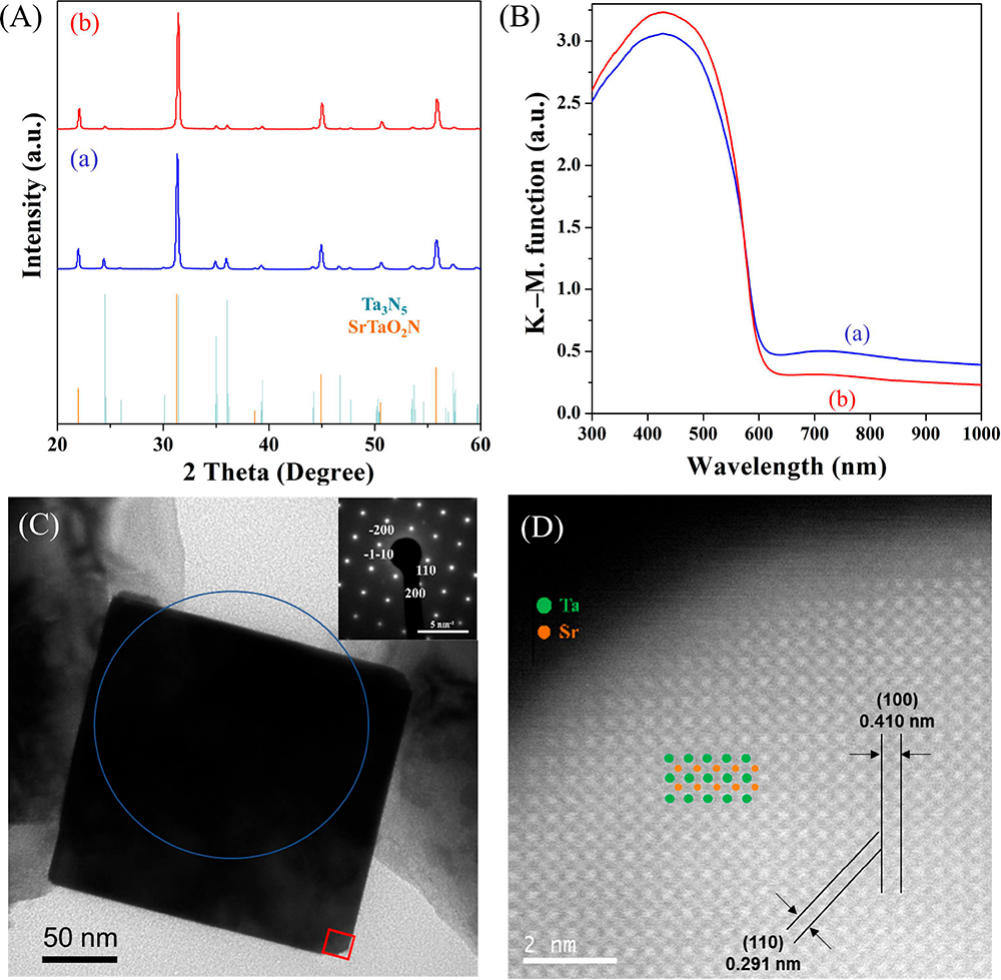
(A) XRD patterns and (B) DRS spectra for (a) SrTaO_2_N(0) and (b) SrTaO_2_N(1). (C) TEM with SAED and (D) ADF-STEM of a cross-section of the SrTaO_2_N(1) sample. The blue circle and red square indicate the areas assessed by SAED and ADF-STEM, respectively.

The selected area electron diffraction (SAED) pattern and the matching of the lattice spacings of SrTaO_2_N(1) with those of a reference SrTaO_2_N crystal structure^12^ suggest the formation of single-crystal SrTaO_2_N nanoparticles (Figures 1C and D). The average size of the cube-like SrTaO_2_N(1) particles was determined to be 124 nm (Figures S1b and S1c). Rod-like particles were also observed in high-resolution transmission electron microscopy (HR-TEM) images. These particles were found not to include Sr species based on analyses by energy dispersive X-ray spectroscopy (EDS) and therefore attributed to Ta_3_N_5_ (Figure S3).

SrTaO_2_N(1) sequentially loaded with IrO_2_ by microwave-assisted heating (denoted as IrO_2(MW)_), Ru by impregnation-H_2_ reduction, and CrO_*y*_ by photodeposition (see the Supporting Information for details) were capable of evolving H_2_ and O_2_ from water simultaneously under visible light (λ > 420 nm) with initial evolution rates of 9.1 and 3.0 μmol h^–1^, respectively (Figure 2A). The deviation from the stoichiometric ratio of the two was due to low quantification accuracy near the detection limit of O_2_ (Figure S4A). The optimal nominal Ir, Ru, and Cr contents were 1, 4, and 4 wt %, respectively (Table S1), while the actual loading proportions were found to be 0.8, 4.0, and 0.6 wt %, respectively, by ICP-AES. The AQY in response to irradiation at 420 ± 30 nm was determined to be 0.34% in the initial stage of the reaction. A slight loss in the OWS activity was observed after three repeated uses of the photocatalyst, after which the performance remained essentially constant with further use. The STH value was measured to be 5–6 × 10^–3^% over the 48-h reaction (Figure S4B). SrTaO_2_N(0) prepared in the absence of NaOH exhibited lower OWS activity because of higher defect density and impurity Ta_3_N_5_ content (Figure S2C). On the other hand, SrTaO_2_N(2) prepared with a larger NaOH amount also exhibited lower OWS activity even though it comprised single-phase SrTaO_2_N. Notably, single-phase SrTaO_2_N was also obtained by using SrCO_3_ instead of NaOH (Figure S5), but it could not split water in this study. These results suggest the importance of controlling the amount of oxygen sources to produce stoichiometric and active SrTaO_2_N. The optimal NaOH/Ta_2_O_5_ molar ratio in the original formulation was unity.

**Figure 2:**
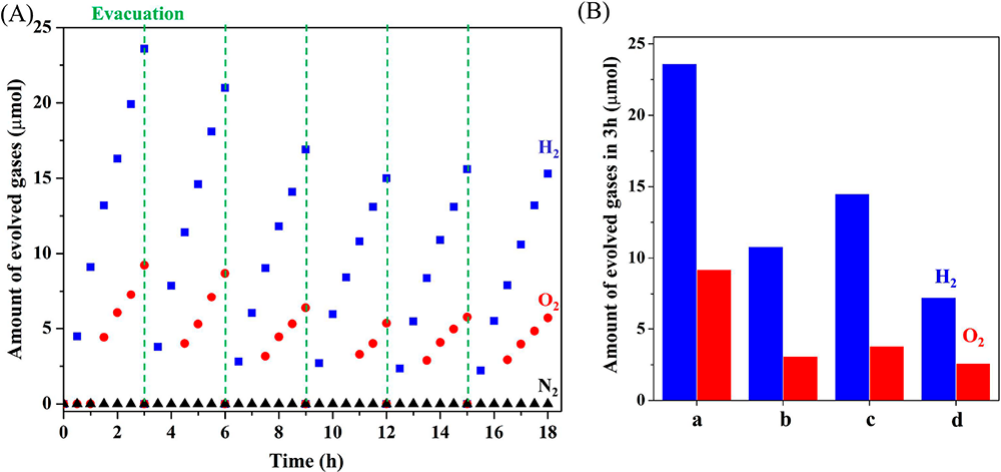
(A) Gas evolution over time using CrO_*y*_/Ru/IrO_2(MW)_/SrTaO_2_N(1) in water under visible light (λ > 420 nm). (B) Amounts of gas products during the 3-h OWS reaction using SrTaO_2_N(1) modified with different cocatalysts: (a) CrO_*y*_/Ru/IrO_2(MW)_, (b) CrO_*y*_/Ru, (c) CrO_*y*_/Ru/IrO_2(AD)_, and (d) IrO_2(AD)_/CrO_*y*_/Ru.

The cocatalysts evidently had a significant effect on the OWS activity. The OWS activity decreased without the loading of IrO_2(MW)_ (Figure 2B). Adsorption of colloidal IrO_2_ (denoted as IrO_2(AD)_) before or after the loading of the CrO_*y*_/Ru cocatalyst was not effective in enhancing OWS although IrO_2(AD)_ effectively promoted the reaction on Y_2_Ti_2_O_5_S_2_,^9^ BaTaO_2_N,^10^ and TaON.^14^ Therefore, an in-depth understanding of the structures and functions of the CrO_*y*_/Ru/IrO_2(MW)_ cocatalyst is imperative.

The annular dark-field scanning TEM (ADF-STEM) images of the CrO_*y*_/Ru/IrO_2(MW)_/SrTaO_2_N(1) specimen in Figure 3 indicate that the Ru particle size in the present samples was around 5 nm. The EDS images show that typical core (Ru species)-shell (Cr species) nanostructures were obtained. The CrO_*y*_ shell capping the noble metal is essential to suppress the reverse reaction during the OWS.^8−10^ The XRD pattern for the Ru/IrO_2(MW)_/SrTaO_2_N(1) suggests the copresence of metallic Ru and RuO_2_ (Figure S6). The latter compound likely resulted from surface oxidation of Ru^0^ nanoparticles, in agreement with our previous results.^14^ On the other hand, the Ir species showed weak signals distributed over the entire SrTaO_2_N(1) particles in STEM-EDS analysis, and their structure was unclear.

**Figure 3:**
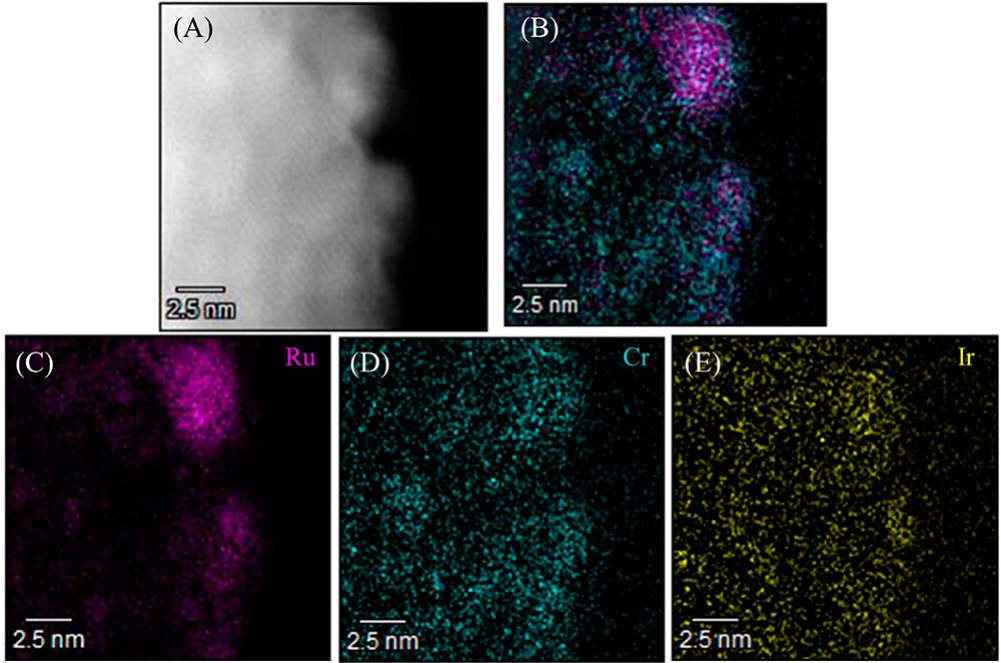
(A) ADF-STEM images and (B-E) STEM-EDS elemental maps of the CrO_*y*_/Ru/IrO_2(MW)_/SrTaO_2_N(1) specimen. (B) Superimposition of Ru and Cr, (C) Ru, (D) Cr, and (E) Ir.

To gain the information about the chemical states, the Ir species loaded on SrTaO_2_N(1) was analyzed by XPS (Figure 4A). The Ir species in IrO_2(MW)_/SrTaO_2_N(1) was attributed to IrO_2_ on the basis of the Ir 4*f*_7/2_ and 4*f*_5/2_ signals at binding energies of 62.2 and 65.2 eV, respectively.^17^ After the H_2_ reduction, the Ir peaks shifted to lower binding energies, indicative of partial reduction to metallic Ir. The fraction of metallic Ir became greater in the presence of Ru to produce the Ru/IrO_2(MW)_ cocatalyst. Concurrently, the fraction of metallic Ru was decreased by the presence of IrO_2(MW)_ compared with the sample without Ir, as shown in the Ru 3*p* XPS analysis (Figure S7). The opposite movements of the Ir and Ru peaks suggest that there were certain electronic interactions between them in the Ru/IrO_2(MW)_ cocatalyst owing to the formation of the RuIr bimetal and RuIrO_2_ bimetallic oxide nanoparticles (denoted as RuIrO_*x*_ for simplicity).^18^ Taking the additional EDS mapping results (Figure S8) into account, it can be concluded that RuO_*x*_, RuIrO_*x*_, and IrO_*x*_ nanoparticles were primarily present on the SrTaO_2_N(1) surface.

**Figure 4:**
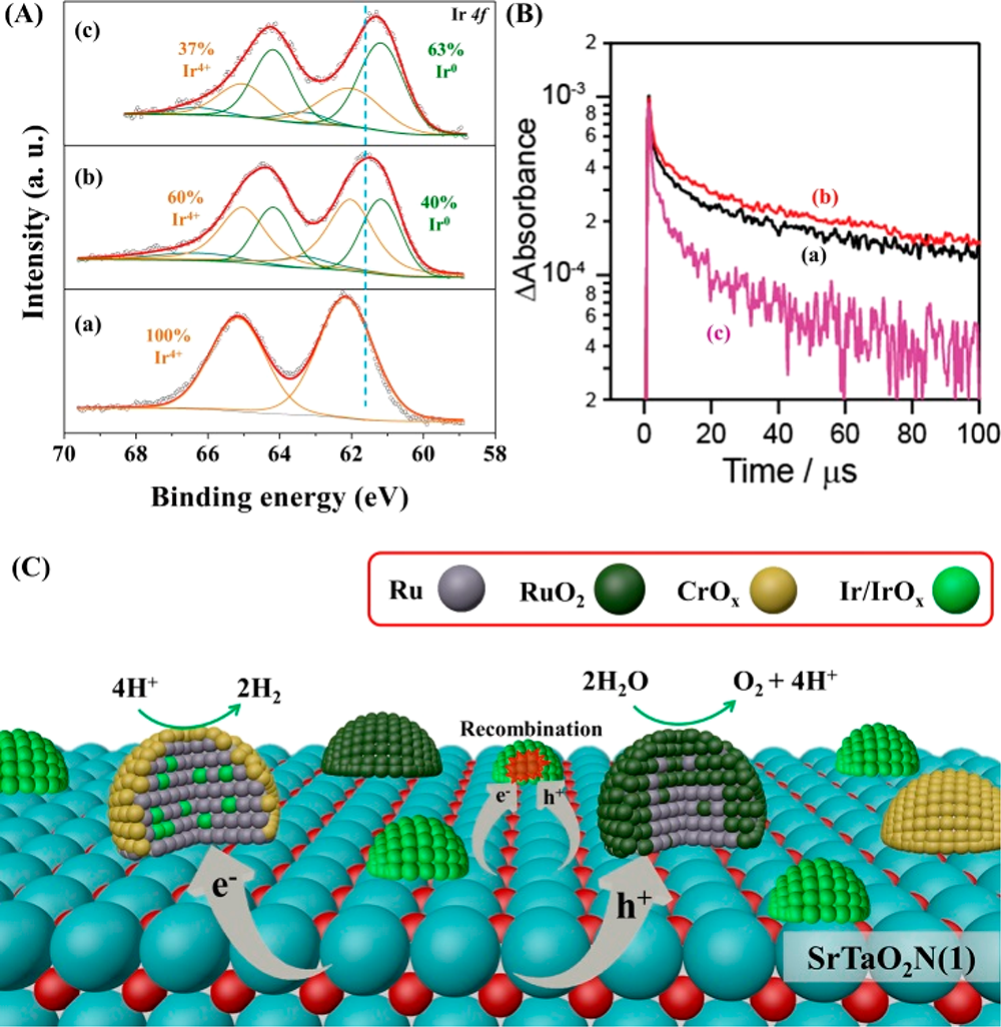
(A) Deconvoluted Ir 4*f* XPS spectra for SrTaO_2_N(1) specimens loaded with (a) IrO_2(MW)_, (b) IrO_2(MW)_ and reduced without loading Ru, and (c) Ru/IrO_2(MW)_. (B) Transient decay profiles of photogenerated electrons probed at 5000 nm of SrTaO_2_N(1) specimens (a) without cocatalysts and loaded with (b) Ru and (c) Ru/IrO_2(MW)_. (C) An illustration showing the structure and dispersion of cocatalysts on the photocatalyst surface together with the dominant charge transfer processes.

The roles of IrO_*x*_, RuO_*x*_, and RuIrO_*x*_ cocatalysts during photocatalytic OWS were further assessed by transient absorption spectroscopy (TAS) (Figure S9).^19^ Probing at a wavelength of 5000 nm (∼0.25 eV) upon band gap excitation of SrTaO_2_N(1) by a 470 nm pump was used to monitor the decay behavior of electrons (Figure 4B). When only RuO_*x*_ was loaded on SrTaO_2_N(1) (i.e., Ru/SrTaO_2_N(1)), the intensity of the absorption at 5000 nm increased, and the decay was notably slower compared with that observed for bare SrTaO_2_N(1). These data demonstrate that Ru/RuO_*x*_ served not only as an HEC but also as an OEC and captured photogenerated holes in SrTaO_2_N(1). These outcomes agree with the observed promotion of the sacrificial HER and OER activities upon loading Ru onto SrTaO_2_N(1) (Figure S10). When Ir was included in RuO_*x*_ (i.e., Ru/IrO_2 (MW)_/SrTaO_2_N(1)) to form the new cocatalyst RuIrO_*x*_, the decay of electrons in SrTaO_2_N(1) was greatly accelerated. Considering the notable enhancement in the sacrificial HER and OER activities compared with the Ru/SrTaO_2_N(1) specimen (Figure S10), this implies that RuIrO_*x*_ captured electrons very efficiently from SrTaO_2_N and worked as an active HEC. Moreover, the TAS results (Figure S11) for IrO_2(MW)_/SrTaO_2_N(1) subjected to the reduction treatment indicate both electrons and holes could be captured by IrO_*x*_, leading to enhanced charge recombination. Collectively, RuIrO_*x*_, having significant electron capture capacity, with a CrO_*y*_ shell worked as an active HEC, while RuO_*x*_ served as a passable OEC (Figure 4C). The IrO_*x*_ species present in minor amounts most likely formed recombination centers that were detrimental to the OWS.

In summary, this work established a SrTaO_2_N-based one-step-excitation OWS system. By reducing the defect density in SrTaO_2_N based on adding NaOH and by the formation of RuIrO_*x*_ cocatalysts that promoted charge separation and surface reactions, this OWS system achieved an STH value of 6.3 × 10^–3^% and an AQY (420 ± 30 nm) of 0.34%. These are one of the highest values yet reported for a perovskite oxynitride photocatalyst with *E*_g_ ≤ 2.1 eV. RuIrO_*x*_ and RuO_*x*_ were found to work as an HEC and OEC, respectively. Further activity improvements are expected by controlling the morphology of the photocatalyst particles to make facet-selective deposition of these cocatalysts feasible.^20^ Controlling the oxygen source during the nitridation and promoting the cocatalyst function by forming bimetallic systems will help the development of other semiconductor/cocatalyst systems for OWS.

## Supporting Information Available

Experimental details; additional characterization and reaction results (PDF)
