# Supplementary material for: Overall Water Splitting by a SrTaO2N-Based Photocatalyst Decorated with an Ir-Promoted Ru-Based Cocatalyst
Source: J Am Chem Soc. 2023 Jan 20;145(7):3839–43. doi: 10.1021/jacs.2c11025 (PMC9952422; doi:10.1021/jacs.2c11025)
Supplement: Supplementary file 1 — ja2c11025_si_001.pdf [file ja2c11025_si_001.pdf]

Overall Water Splitting by a $SrTaO_2N$-Based Photocatalyst Decorated with an Ir-Promoted Ru-Based Cocatalyst

Kaihong Chen,[a,||] Jiadong Xiao,[a,||] Junie Jhon M. Vequizo,[a] Takashi Hisatomi,[a,b] Yiwen Ma,[a] Mamiko Nakabayashi,[c] Tsuyoshi Takata,[a] Akira Yamakata,[d] Naoya Shibata,[c] Kazunari Domen[a,e]*

[a] Research Initiative for Supra-Materials, Interdisciplinary Cluster for Cutting Edge Research, Shinshu University, Nagano-shi, Nagano 380-8553, Japan.

[b] PRESTO, JST, 4-17-1 Wakasato, Nagano-shi, Nagano 380-8553, Japan.

[c] Institute of Engineering Innovation, The University of Tokyo, 2-11-16 Yayoi, Bunkyo-ku, Tokyo 113-8656, Japan.

[d] Graduate School of Natural Science & Technology, Okayama University, 3-1-1 Tsushima-naka, Okayama 700-8530, Japan.

[e] Office of University Professors, The University of Tokyo, 2-11-16 Yayoi, Bunkyo-ku, Tokyo 113-8656, Japan.

Experimental Procedures

Chemicals. Ta_2O_5 (Rare Metallic Co., Ltd.), $SrCl_2$, NaOH (Fujifilm Wako Pure Chemical Co.), $RuCl_3 \cdot 3H_2O$ (Wako Pure Chemical Industries; 99.9%), K_2CrO_4 (Kanto Chemical Co.; 99.9%) and $IrCl_3 \cdot nH_2O$ (TCI, 50.0-56.0% as Ir) were used as received. $IrCl_3 \cdot nH_2O$ was handled as $IrCl_3 \cdot 3H_2O$. Deionized water was used for all syntheses and is simply referred to as water herein.

Characterization. X-ray diffraction (XRD) patterns were acquired using a Rigaku MiniFlex 300 powder diffractometer with a Cu Kα radiation source. Diffuse reflectance spectroscopy (DRS) was conducted using an ultraviolet-visible-near infrared spectrometer (V-670, JASCO). X-ray photoelectron spectroscopy (XPS) was performed with a PHI Quantera II spectrometer, employing an Al Kα radiation source. All binding energies were corrected based on the C 1*s* peak (284.8 eV) as a reference. Scanning electron microscopy (SEM), high-resolution transmission electron microscopy (HR-TEM) and (scanning) transmission electron microscopy ((S)TEM) images together with energy-dispersive X-ray spectroscopy (EDS) elemental maps were acquired using Hitachi HD2300A, JEOL JEM-2800 and JEOL JEM-ARM200F Thermal FE STEM (Cs STEM) instruments, respectively. Inductively coupled plasma-atomic emission spectroscopy (ICP-AES) analyses were performed with a Shimadzu ICPS-8100 instrument. The oxygen and nitrogen contents of the sample were determined using an oxygen-nitrogen combustion analyzer (Horiba, EMGA-620W). Transient absorption (TA) data were obtained using a pump-probe Nd:YAG laser system (Continuum, Surelite I; duration: 6 ns) with custom-built spectrometers.[1-3] The photoexcited electrons were probed at 2000 cm^{-1} (5000 nm) using an infrared light beam emitted by a $MoSi_2$ coil that was passed through a photocatalyst sample formed into a film. The transmitted IR beam was subsequently sent to a monochromatic grating spectrometer and detected by a mercury-cadmium-telluride detector (Kolmar). The output electric signal was amplified using an alternating current coupled amplifier (Stanford Research Systems, SR560, bandwidth: 1 MHz). The time resolution of the spectrometer was limited to 1 μs by the bandwidth of the amplifier. TA spectra were acquired by scanning the probe energy from 5000 cm^{-1} (2000 nm, 0.62 eV) to 1200 cm^{-1} (8333 nm, 0.15 eV). Data acquisition was performed by accumulating 3000 TA signals/responses to generate each decay curve. A 470 nm laser pulse with a fluence of 0.12 mJ $pulse^{-1}$ and a frequency of 1 Hz was used to excite the charge carriers in $SrTaO_2N$(1) specimens with and without cocatalysts. These analyses were carried out in a N_2 atmosphere at a pressure of 20 Torr. Each sample was prepared by dispersing the powdered photocatalyst in water and then drop-casting this dispersion on a CaF_2 substrate followed by air drying overnight to obtain a film with a density of approximately 1.6 mg cm^{-2}.

Synthesis of $SrTaO_2N$(*n*). $SrTaO_2N$(*n*) was synthesized by the nitridation of $SrCl_2$, Ta_2O_5 and NaOH combined in a 4:1:*n* molar ratio, where *n* represents the

$NaOH/Ta_2O_5$ molar ratio. Taking the synthesis of $SrTaO_2N(1)$ as an example. The $SrTaO_2N(1)$ samples were prepared using a one-pot flux-assisted nitridation approach. $SrCl_2$ (0.86 g), Ta_2O_5 (0.6 g) and NaOH (0.054 g) were mixed in a mortar. This mixture was then transferred into an alumina crucible and nitrided at 950 °C for 5 h under a NH_3 flow at a rate of 200 mL min^{-1}. After cooling naturally to room temperature, each sample was washed several times with hot water and then dried at 40 °C for 8 h. For comparison purposes, a $SrTaO_2N(0)$ sample was also synthesized using the same procedure but without adding NaOH. $SrTaO_2N$ was also synthesized by the same procedure with $SrTaO_2N(2)$ except the replacing NaOH with $SrCO_3$, and the product was denoted as $SrTaO_2N(SrCO_3)$. Note that the molar ratio of $SrCO_3/Ta_2O_5$ in $SrTaO_2N(SrCO_3)$ was two.

Cocatalyst loading. The $SrTaO_2N(1)$ (170 mg) was dispersed in water (15 mL) followed by the addition of an appropriate amount of an aqueous $IrCl_3$ solution (2 mg/ml). This mixture was then heated in a microwave reactor (Monowave 200, Anton Paar) at 150 °C for 10 min.[4,5] The material was recovered by filtration and then dried at 40 °C for 0.5 h. Ru was added to the resulting $IrO_{2(MW)}/SrTaO_2N(1)$ powder using a conventional impregnation-thermal reduction process. This process involved heating at 250 °C for 1 h under a flow of a H_2 and N_2 mixture (H_2: 20 mL min^{-1}; N_2: 200 mL min^{-1}). CrO_y was subsequently photodeposited in an aqueous methanol solution (13 vol%) with visible light irradiation ($\lambda > 420$ nm) for 3 h.

Photocatalytic overall water splitting reactions and half reactions. All photocatalytic reactions were carried out at 15 °C under Ar at a background pressure of 5 kPa in a Pyrex reaction vessel connected to a closed gas circulation system. In a typical OWS experiment, a 150 mg quantity of the $CrO_y/Ru/IrO_{2(MW)}$-loaded photocatalyst was dispersed in 150 mL of ultrapure water adjusted to a pH of 8 by adding a 0.1 M NaOH solution. After completely removing air from the reaction slurry by evacuation, the suspension was irradiated with a 300 W xenon lamp equipped with a dichroic mirror and an L42 cut-off filter to emit visible light ($\lambda > 420$ nm). The evolved gaseous products were analyzed by an integrated online gas chromatography system consisting of a GC-8A chromatograph (Shimadzu) equipped with 5 Å molecular sieve columns and a thermal conductivity detector, using Ar as the carrier gas. The procedures employed for the HER and OER half reactions were similar except that an aqueous methanol solution (13 vol%) was used as the sacrificial reagent in the HER reaction and a $AgNO_3$ aqueous solution (10 mM) was used as the sacrificial reagent together with 150 mg La_2O_3 as a pH buffer in the OER reaction.

Apparent quantum yield (AQY) calculations. The AQY for the OWS reaction was calculated as

$$\mathrm{AQY}(\%) = \frac{[2 \times n(\mathrm{H_2}) + 4 \times n(\mathrm{O_2})]}{2} \frac{1}{n(\mathrm{photons})} \times 100$$

where $n(H_2)$, $n(O_2)$, and n(photons) represent the quantities of evolved H_2 and O_2 molecules and incident photons, respectively. The light source was the same as that

employed for the OWS reactions except that a 420 nm bandpass filter was used. The number of incident photons was determined with an LS-100 grating spectroradiometer (EKO Instruments Co., Ltd.).

Solar-to-hydrogen (STH) energy conversion efficiency calculations. The STH values were calculated using the relationship

$$\mathrm{STH}(\%) = [r(\mathrm{H_2}) \times \Delta G_\mathrm{r}]/(P \times S) \times 100$$

where $r(H_2)$, ΔG_r, P and S denote the H_2 evolution rate during the OWS reaction, the Gibbs energy for the water splitting reaction at the standard condition (237 kJ mol^{-1}), the energy intensity of the AM 1.5G solar irradiation (100 $mW \cdot cm^{-2}$) and the irradiated sample area, respectively.

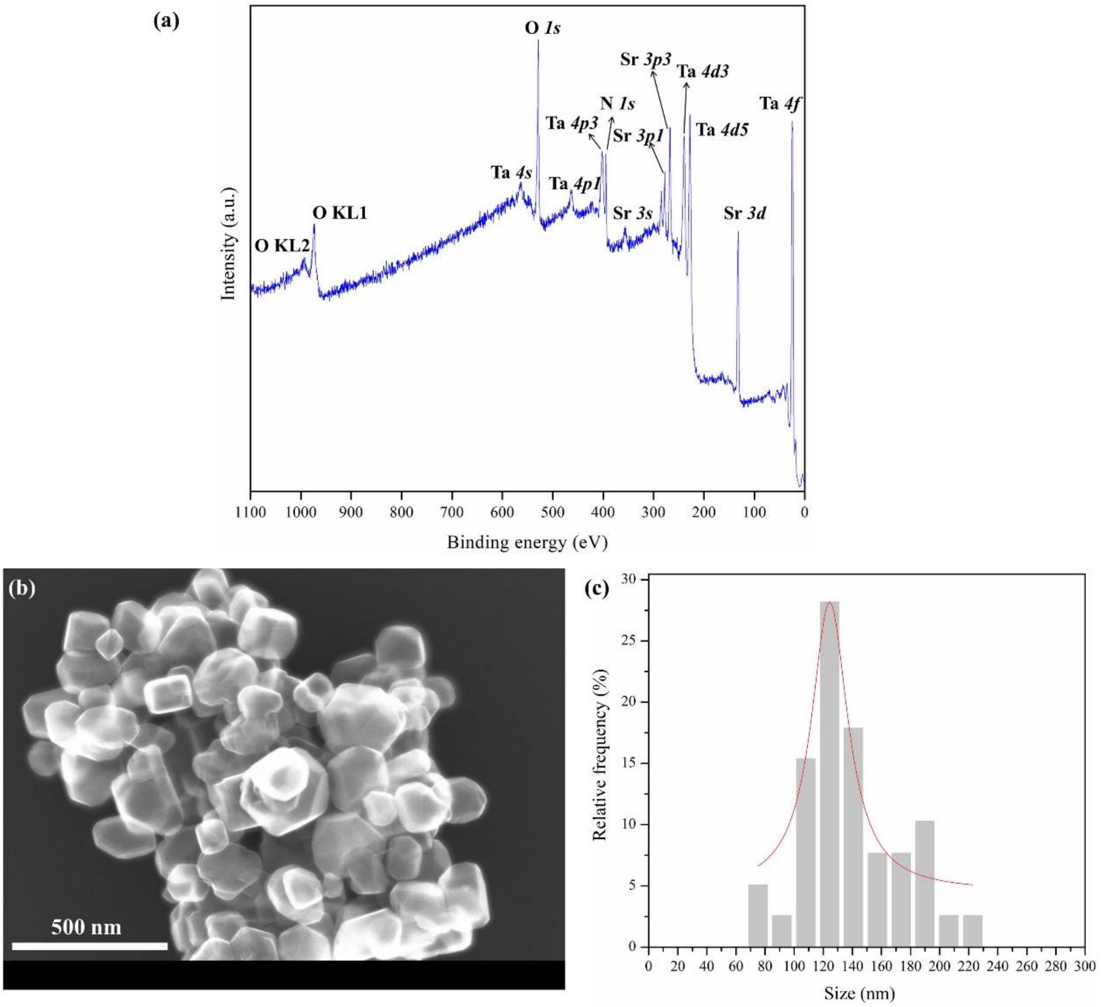

Figure S1. (a) XPS spectrum, (b) FE-SEM image and (c) particle size distribution for $SrTaO_2N$(1).

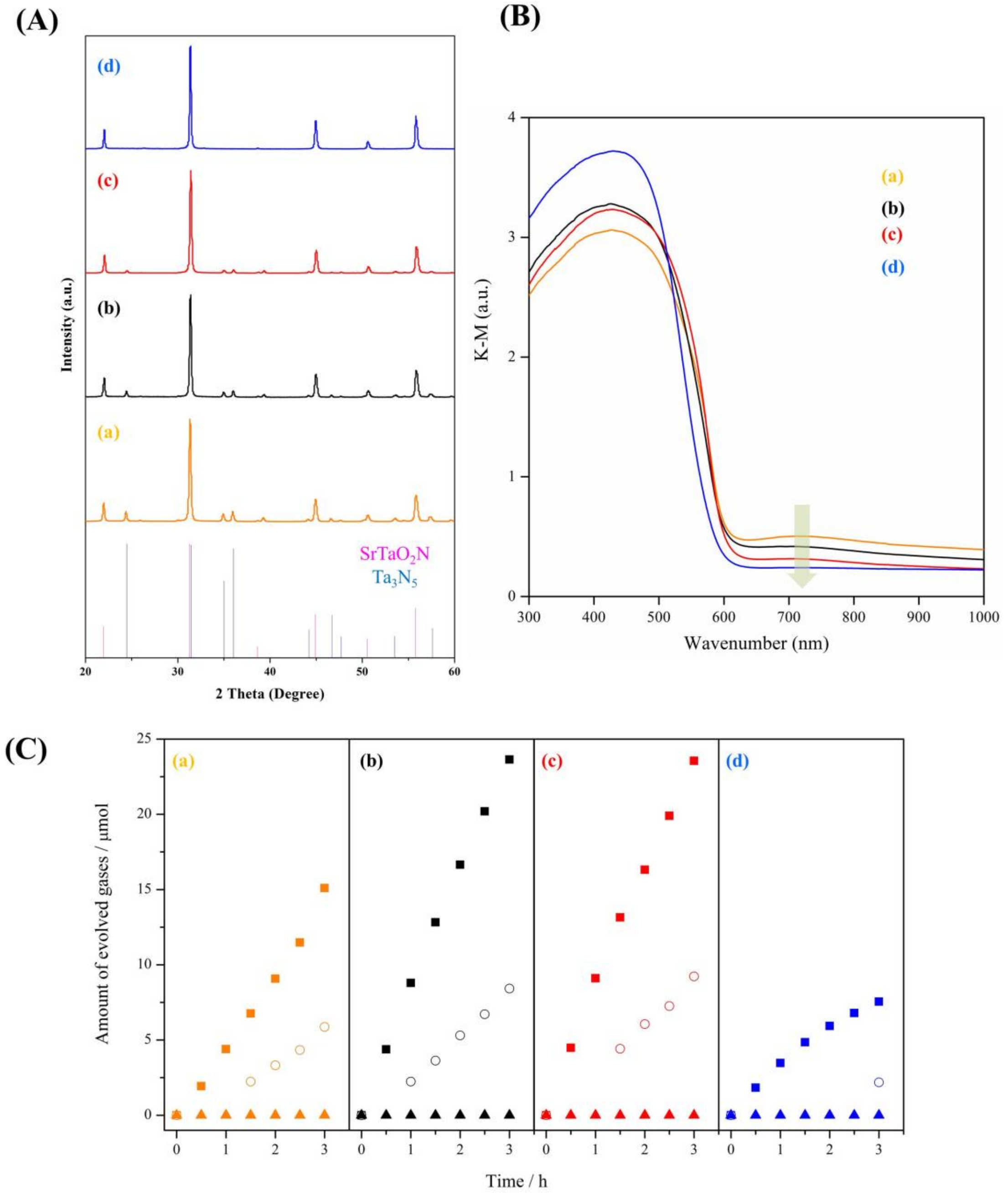

Figure S2. (A) XRD patterns, (B) DRS data and (C) OWS activity for $SrTaO_2N$(*n*) specimens synthesized using varying amounts of NaOH. The value of *n* are (a) 0, (b) 0.4, (c) 1, and (d) 2, respectively. The squares, circles, and triangles in (C) are represented H_2, O_2, and N_2, respectively.

Note: A small peak ascribed to defects in the Ta_3N_5 by-product can be discerned at 700 nm in Figure S2B.

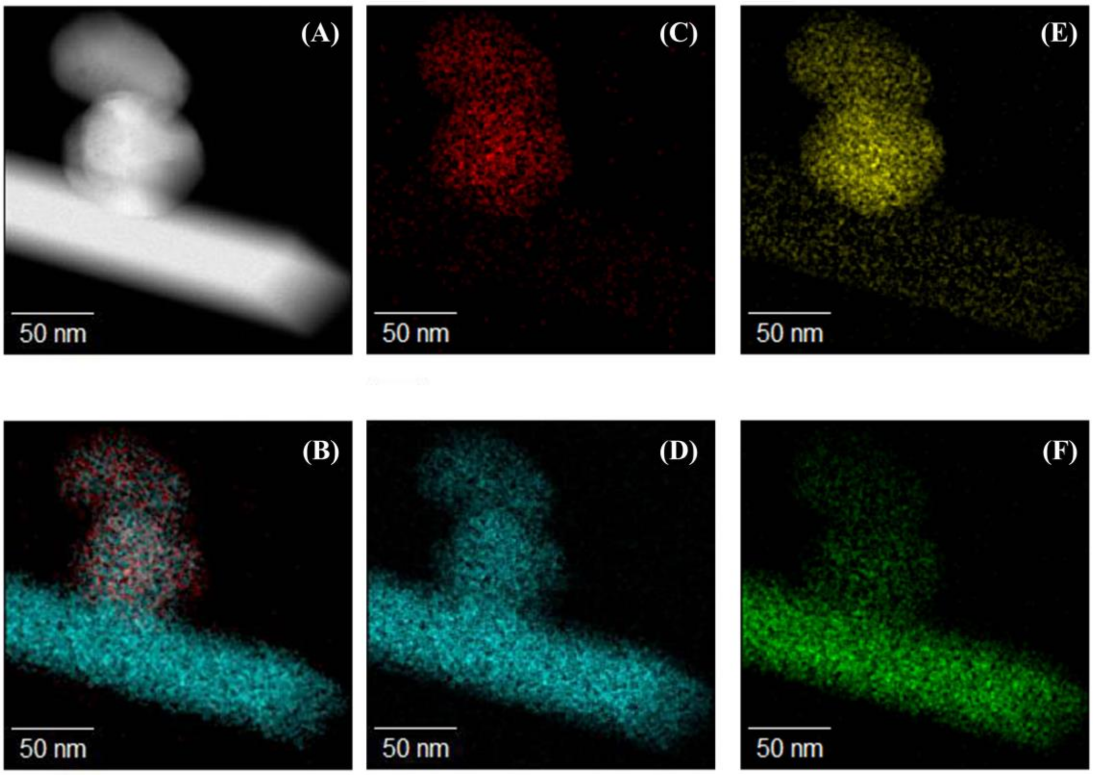

Figure S3. (A) ADF-STEM images, (B) overlay images of STEM-EDS elemental maps of (C) Sr, (D) Ta, (E) O, and (F) N in $SrTaO_2N$(1) specimen.

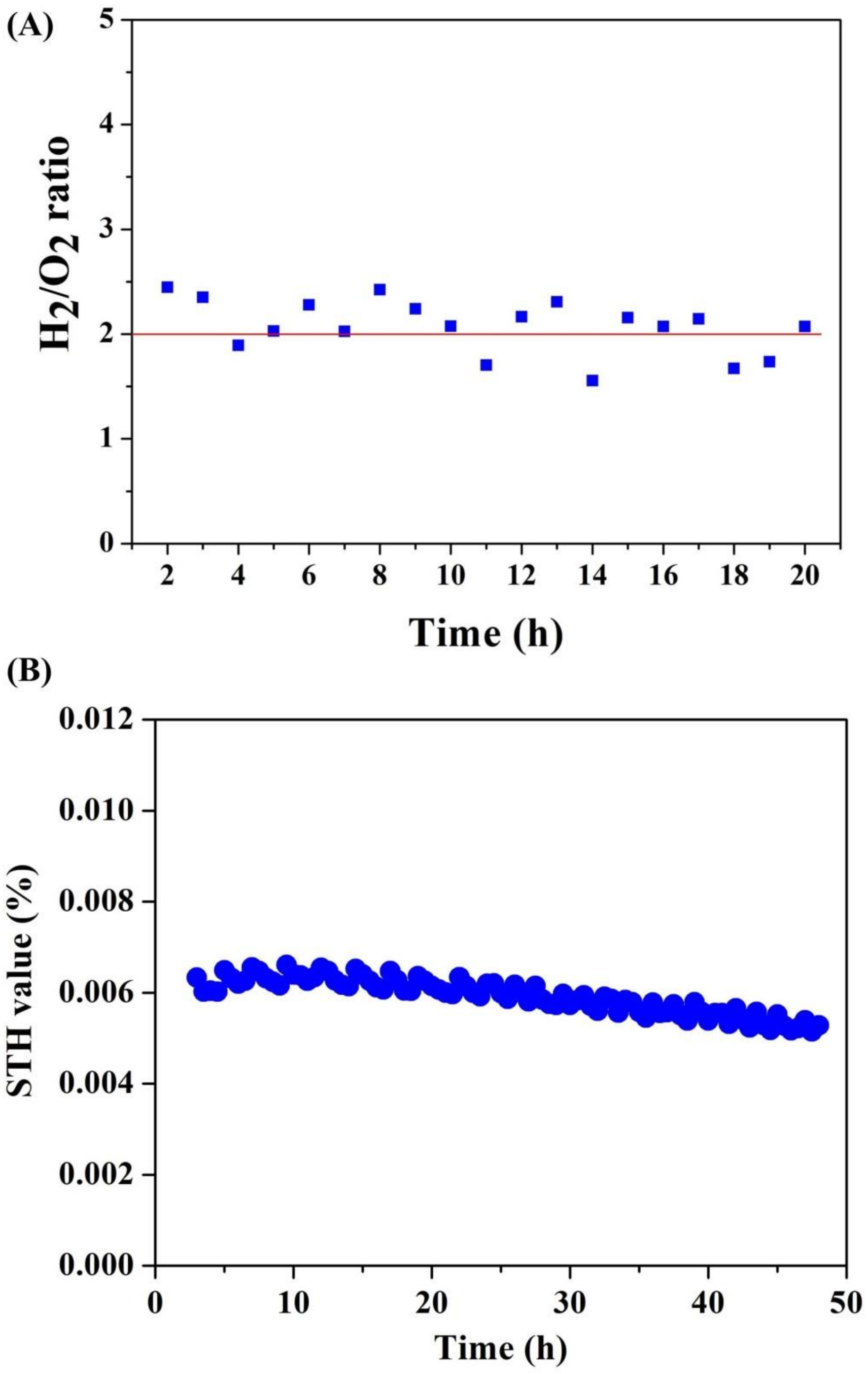

Figure S4. (A) The H_2/O_2 ratio calculated from the incremental amounts of H_2 and O_2 at each sampling (hourly) during a 20-h reaction using $CrO_y/Ru/IrO_{2(MW)}/SrTaO_2N(1)$ in water under visible light ($\lambda > 420$ nm). (B) STH values obtained over time from $CrO_y/Ru/IrO_{2(MW)}/SrTaO_2N(1)$ in water under AM 1.5 G and Ar background pressure of 5 kPa.

Table S1. OWS activities of $SrTaO_2N$(1) specimens loaded with different cocatalyst amounts[a]

Entry	Ir amount (wt%)	Ru amount (wt%)	Cr amount (wt%)	Gas evolution in 3 h (μmol)[b]		
				H_2	O_2	N_2
1	1	4	4	23.6	9.2	N.D.
2	0	4	4	10.8	3.1	N.D.
3	0.5	4	4	11.4	4.0	N.D.
4	2	4	4	12.7	4.6	N.D.
5	1	0	0	N.D.	N.D.	N.D.
6	1	0	1	N.D.	N.D.	N.D.
7	1	2	2	5.4	N.D.	N.D.
8	1	6	6	11.7	4.0	N.D.
9	1	4	0	10.4	N.D.	N.D.
10	1	4	2	10.2	3.8	N.D.
11	1	4	8	16.9	6.9	N.D.

[a] Reaction condition: 150 mg photocatalyst with cocatalysts; 150 mL H_2O (pH = 8; adjusted by NaOH (aq.)); Xe lamp ($\lambda \geq 420$ nm); 5 kPa; [b] N.D.: Not Detected.

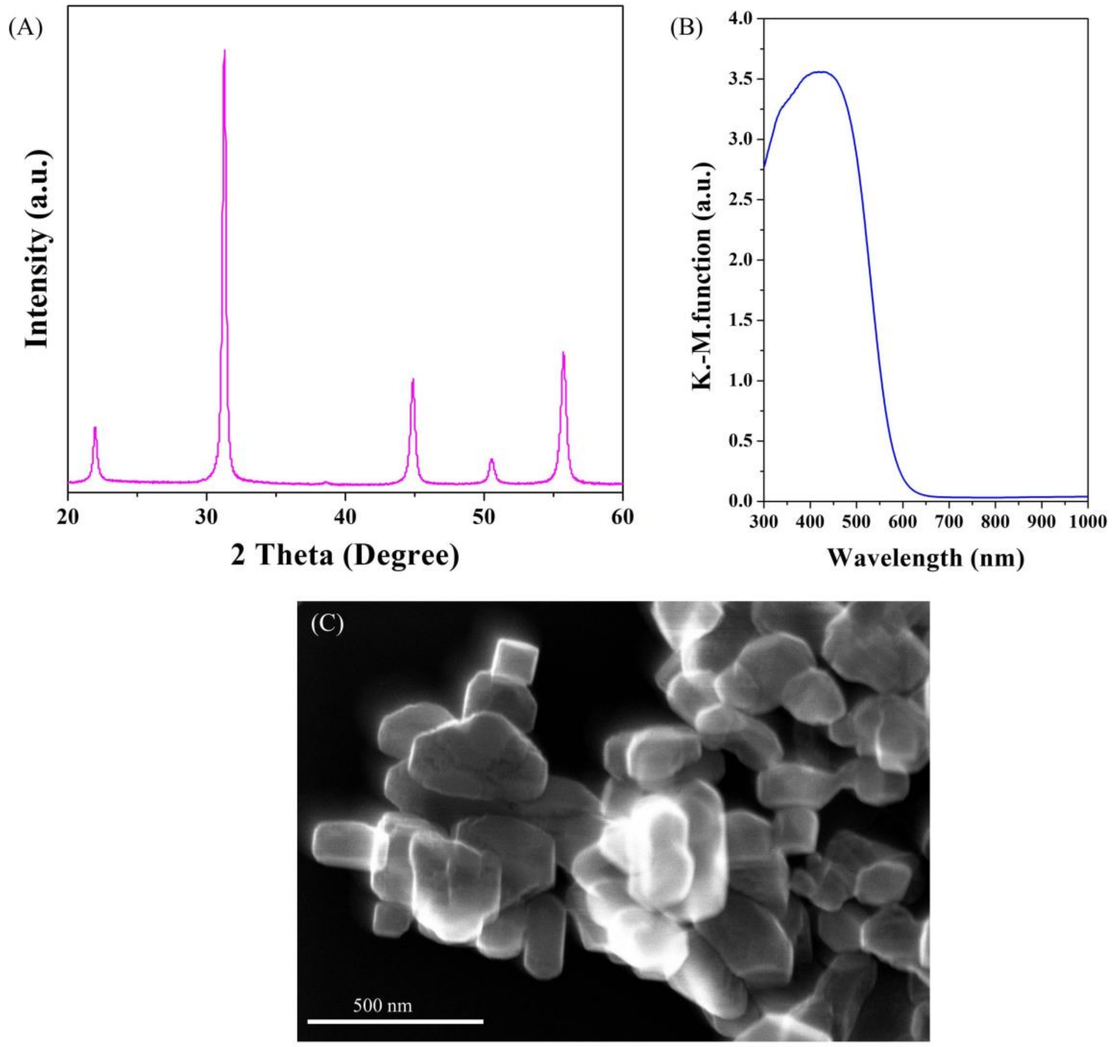

Figure S5. The (A) XRD pattern, (B) DRS spectrum, and (C) FE-SEM image of $SrTaO_2N(SrCO_3)$.

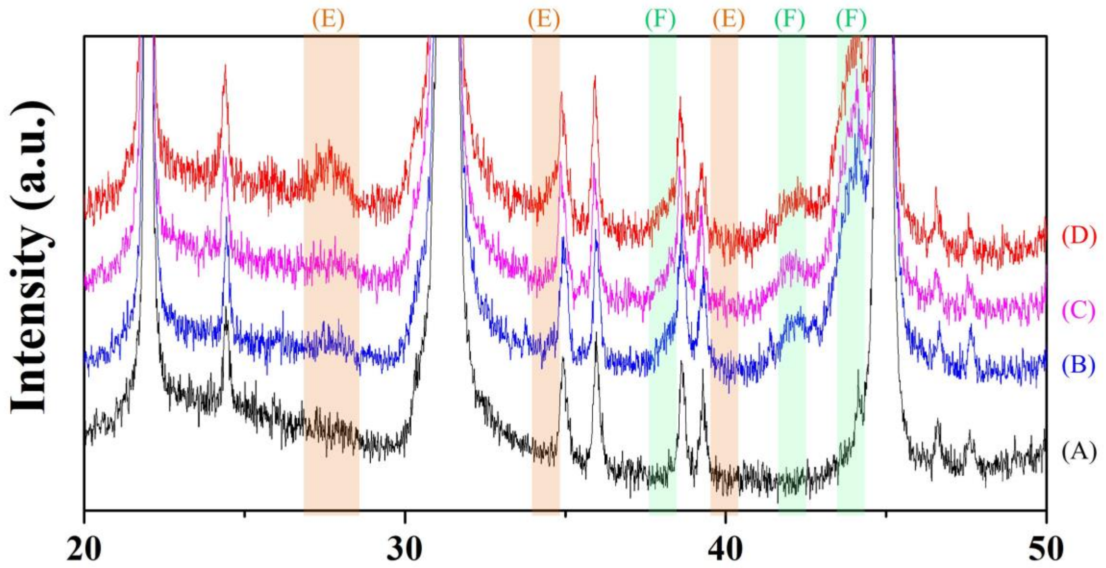

Figure S6. XRD patterns obtained from (A) bare $SrTaO_2N$(1), (B) microwave-treated $SrTaO_2N$(1) loaded with Ru, and $SrTaO_2N$(1) loaded with (C) Ru and (D) Ru/$IrO_{2(MW)}$. The XRD standard peaks position of (E) RuO_2 and (F) Ru were also shown.

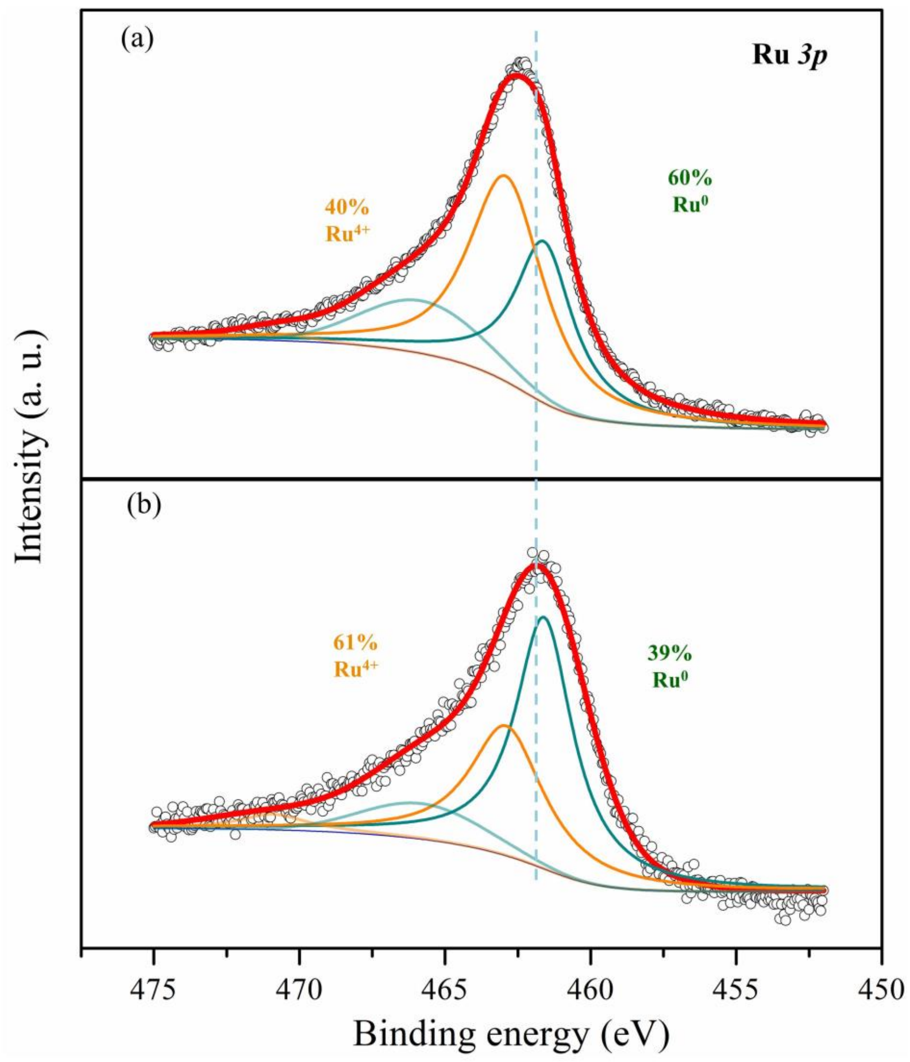

Figure S7. Deconvoluted Ru 3*p* XPS spectra of $SrTaO_2N(1)$ specimens loaded with (a) Ru and (b) $Ru/IrO_{2(MW)}$.

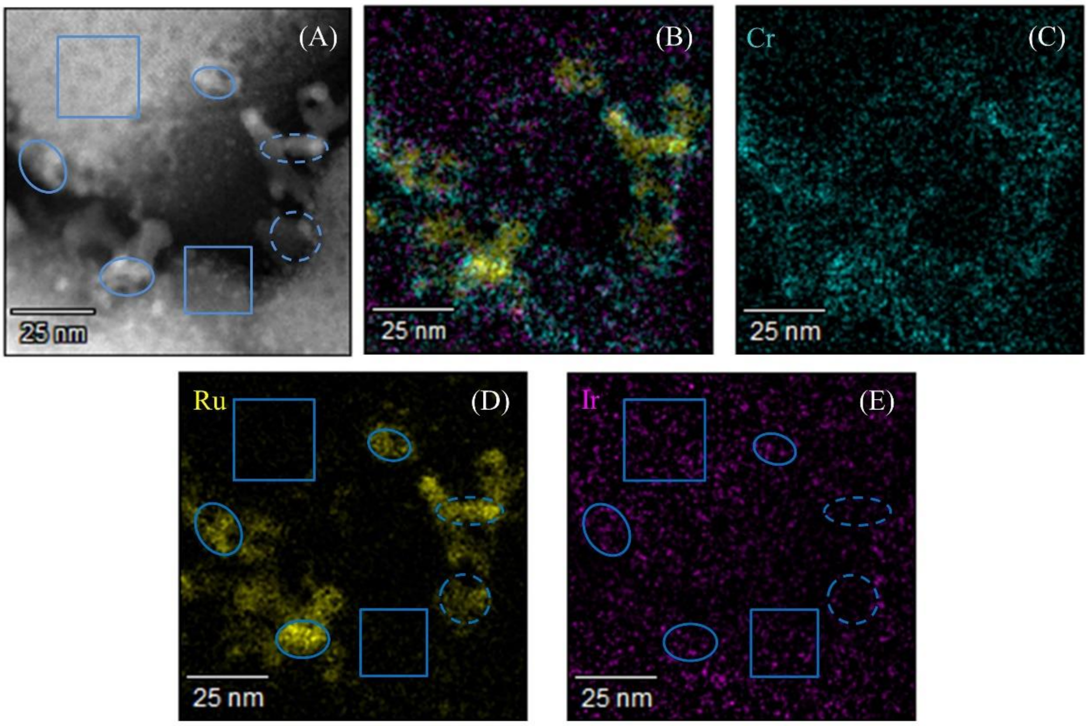

Figure S8. (A) ADF-STEM images and (B-E) STEM-EDS elemental maps of a cross-sectional $CrO_y/Ru/IrO_{2(MW)}/SrTaO_2N(1)$ sample. (B) superimposition of Cr, Ru, and Ir, (C) Cr, (D) Ru, and (E) Ir. Solid and dotted circles and solid square represent $RuIrO_x$, RuO_x, and IrO_x respectively.

Note: The particles indicated by solid circles in Figure S8 may represent $RuIrO_x$, while those enclosed with dotted circles were most likely RuO_x as minimal Ir was detected in these areas. Moreover, there was no Ru detected in solid square, so that only IrO_x existed in these areas. In addition, in Figure 3, Cr was detected around the Ru. These observations support the formation of CrO_y shell on the $RuIrO_x$ core.

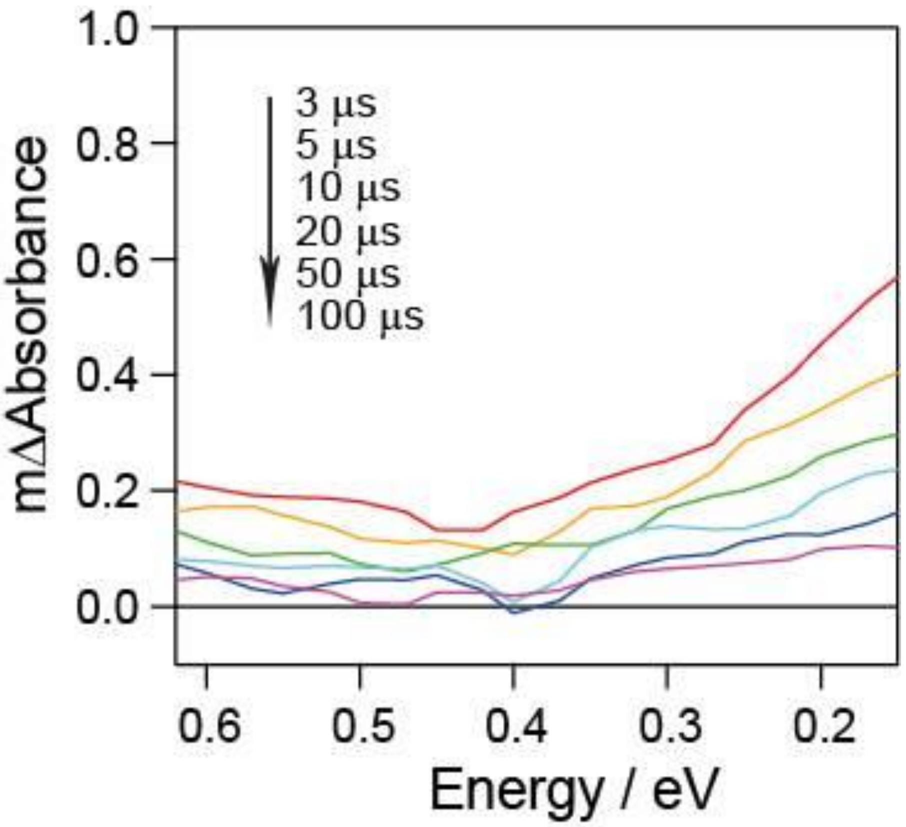

Figure S9. Mid-IR transient absorption spectra of photocarriers in $SrTaO_2N$(1) acquired 3-100 μs after excitation using 470 nm laser pulses (0.12 mJ $pulse^{-1}$, repetition rate: 1 Hz) under N_2 (20 Torr).

Note: As shown in Figure S9, the mid-IR transient absorption spectra generated upon band-gap excitation of $SrTaO_2N$(1) by a 470 nm pump exhibited structureless absorption characteristics becoming stronger with decreasing the probe light energy. These results are attributed to shallowly trapped and/or mobile electrons.

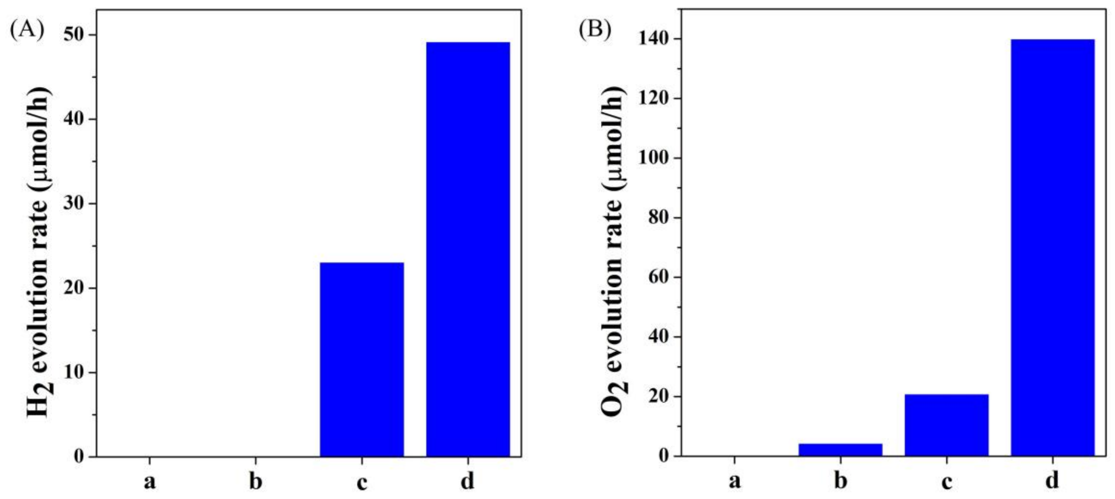

Figure S10. (A) Hydrogen and (B) oxygen evolution activity of $SrTaO_2N$(1) (a) without cocatalyst and loaded with (b) $IrO_{2(MW)}$, (c) Ru, and (d) Ru/$IrO_{2(MW)}$. The reaction solutions used for (A) and (B) were aqueous methanol and silver nitrate solutions, respectively.

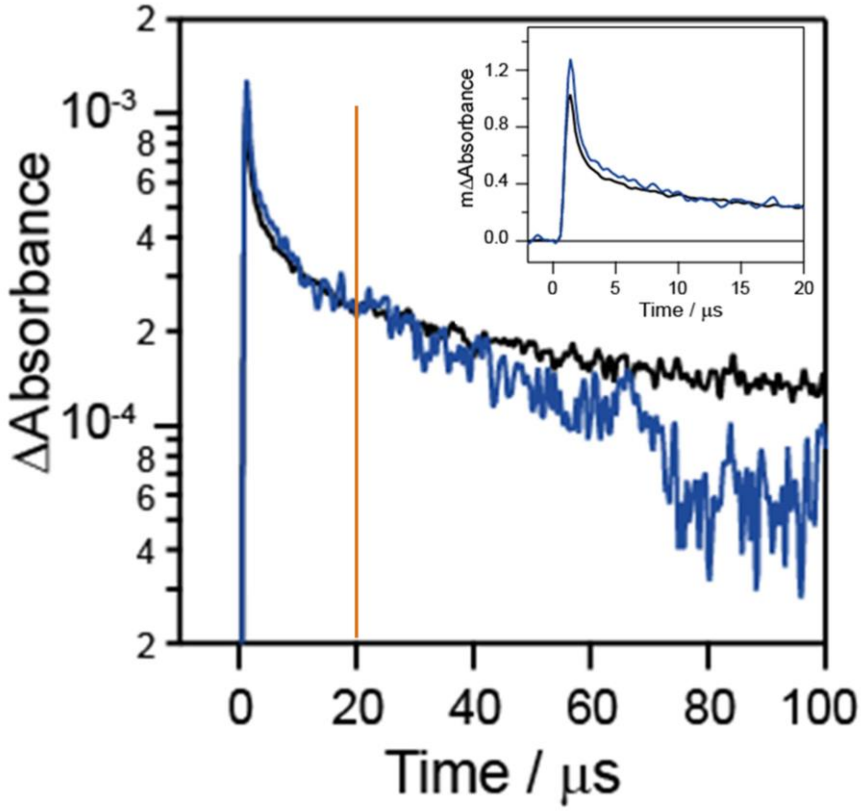

Figure S11. Transient decay profiles of electrons probed at 5000 nm in bare $SrTaO_2N(1)$ (black) and reduced $IrO_{2(MW)}/SrTaO_2N(1)$ (blue). The samples were excited by 470 nm laser pulses (0.12 mJ pulse^{-1}, repetition rate: 1 Hz) under N_2 (20 torr). The inset shows the profiles over the range of 0-20 µs.

Note: The TAS signal intensity for $IrO_{2(MW)}/SrTaO_2N(1)$ subjected to the reduction treatment was higher than that for bare $SrTaO_2N(1)$ over the range of 0-20 µs, indicating that the electron population was increased as a result of hole capture by IrO_x (Figure S11). However, past 20 µs, the signal produced by the reduced $IrO_{2(MW)}/SrTaO_2N(1)$ decreased more rapidly than that for the bare material.

REFERENCES

(1) Vequizo, J. J. M.; Matsunaga, H.; Ishiku, T.; Karnimura, S.; Ohno, T.; Yamakata, A. Trapping-Induced Enhancement of Photocatalytic Activity on Brookite TiO_2 Powders: Comparison with Anatase and Rutile TiO_2 Powders. *ACS Catal.* **2017**, *7*, 2644-2651.

(2) Yamakata, A.; Vequizo, J. J. M.; Ogawa, T.; Kato, K.; Tsuboi, S.; Furutani, N.; Ohtsuka, M.; Muto, S.; Kuwabara, A.; Sakata, Y. Core-Shell Double Doping of Zn and Ca on beta-Ga_2O_3 Photocatalysts for Remarkable Water Splitting. *ACS Catal.* **2021**, *11*, 1911-1919.

(3) Xiao, J. D.; Vequizo, J. J. M.; Hisatomi, T.; Rabeah, J.; Nakabayashi, M.; Wang, Z.; Xiao, Q.; Li, H. H.; Pan, Z. H.; Krause, M.; Yin, N.; Smith, G.; Shibata, N.; Bruckner, A.; Yamakata, A.; Takata, T.; Domen, K. Simultaneously Tuning the Defects and Surface Properties of Ta_3N_5 Nanoparticles by Mg-Zr Codoping for Significantly Accelerated Photocatalytic H_2 Evolution. *J. Am. Chem. Soc.* **2021**, *143*, 10059-10064.

(4) Akiyama, S.; Nakabayashi, M.; Shibata, N.; Minegishi, T.; Asakura, Y.; Abdulla-Al-Mamun, M.; Hisatomi, T.; Nishiyama, H.; Katayama, M.; Yamada, T.; Domen, K.; Highly efficient water oxidation photoanode dade of surface modified $LaTiO_2N$ particles. *Small* **2016**, *12*, 5468-5476.

(5) Zhou, F.; Liu, Q.; Gu, J.; Zhang, W.; Zhang, D.; A facile low-temperature synthesis of highly distributed and size-tunable cobalt oxide nanoparticles anchored on activated carbon for supercapacitors. *J. Power Sources* **2015**, *273*, 945-953.
